# Pinoresinol enhances oral barrier integrity and function in human buccal cell monolayers

**DOI:** 10.1371/journal.pone.0331242

**Published:** 2025-09-08

**Authors:** Soo-Bin Shin, Young-Min Kim, Ha-Young Park

**Affiliations:** 1 Advanced Radiation Technology Institute, Korea Atomic Energy Research Institute, Jeongeup, Republic of Korea; 2 Department of Integrative Food, Bioscience and Biotechnology, Chonnam National University, Gwangju, Republic of Korea; Eötvös Loránd Research Network Biological Research Centre, HUNGARY

## Abstract

The oral epithelial barrier plays a crucial role in maintaining oral health by protecting against microbial invasion and mechanical stress while regulating selective permeability. Disruption of this barrier contributes to inflammation and the development of oral diseases such as gingivitis and periodontitis. Pinoresinol, a lignan with antioxidant, antimicrobial, and anti-inflammatory properties, has demonstrated health benefits in systemic diseases; however, its effects on oral epithelial barrier integrity remain unclear. This study investigated the potential of pinoresinol to enhance oral epithelial barrier function using TR146 cell monolayers. Pinoresinol treatment significantly reduced apparent permeability and increased transepithelial electrical resistance, indicating improved barrier integrity. Additionally, pinoresinol upregulated the expression of tight junction-associated molecules, including occludin, MarvelD3, claudin-1, and claudin-3, at both the mRNA and protein levels, enhanced their localization as shown by immunofluorescence. These findings suggest that pinoresinol reinforces the oral epithelial barrier and may help prevent barrier dysfunction associated with oral diseases.

## Introduction

The oral cavity is a unique environment, constantly exposed to mechanical, chemical, and biological stimuli throughout an individual's life [1]. The oral mucosa, which covers the oral cavity, plays a dual role: it protects underlying tissues from mechanical damage while also serving as an important barrier and entry point for food, microorganisms, and airborne particles destined for the gastrointestinal system [2]. Structurally, the oral mucosa consists of a middle layer of squamous epithelium and an underlying connective tissue layer known as the lamina propria [3]. The oral epithelium acts as the primary defense mechanism, isolating the host from environmental hazards and protecting against pathogens, foreign bodies, and mechanical stress [4]. Oral epithelial cells adhere to a tightly regulated differentiation process that ensures tissue integrity by forming structural proteins critical to barrier function [5]. However, when this integrity is compromised due to mutations in certain proteins or dysregulated signaling pathways, the barrier can become impaired and vulnerable to disease. Such damage often triggers an inflammatory response, where cytokines and chemokines are released from connective tissue cells, recruiting immune cells and exacerbating tissue infiltration and damage [6,7]. Building on the relationship between epithelial barrier dysfunction and disease, recent studies have shown that compromised oral epithelial integrity and defective keratinocyte differentiation may contribute to the development of oral squamous cell carcinoma (OSCC), which originates from epithelial basal cells [1]. This principle is not limited to oral epithelium, as epithelial barrier disruption is also implicated in conditions such as inflammatory bowel disease and skin allergies [8]. Tight junctions (TJs) perform an important barrier function between epithelial cells and are critical in maintaining the integrity of the oral epithelium [9]. TJs are formed by the assembly of multiple specific proteins near the apical portion of the lateral membrane and are linked to the cytoskeleton. The TJ is a highly dynamic region, and its permeability can change in response to external and intracellular stimuli [10]. TJs, including claudins and occludin, are essential membrane proteins important for sealing the gaps between epithelial cells, ensuring the integrity of the barrier [11]. TJs interact with the zonula occludens (ZO) family of skeletal proteins through their cytoplasmic domains and play an essential role in TJ assembly. In addition to claudins, other membrane proteins localize to TJs, such as the TJ-associated MARVEL domain-containing proteins (TAMPs: occludin, trichellulin, and marvelD3) and junctional adhesion molecules (JAMs) within the immunoglobulin superfamily [12,13]. TJs create a selective barrier between cells, controlling the passage of ions, small molecules, and large macromolecules. When TJs are disrupted or weakened by inflammatory stimuli, mechanical stress, or pathological conditions, selective permeability is impaired, resulting in the increased passage of unwanted substances through the epithelial layer [14]. Epithelial permeability can be predicted from pericellular ion fluxes, and barrier integrity correlates with TJ protein expression [15]. TR146 cells derived from neck nodal metastases of human buccal carcinoma are used for this permeability assessment and are suitable for studying the transport of selected markers and drugs [16]. A diet rich in polyphenols, found in foods such as green tea, coffee, berries, grapes, and various fruits and vegetables, helps maintain epithelial barrier function and enhance TJ-related protein expression. Polyphenols, which are bioactive compounds classified as phenolic acids, flavonoids, anthocyanins, stilbenes, and lignans, are known for their anti-inflammatory and antioxidant effects and support protection and strengthening of the epithelial barrier [17–19]. Polyphenols, such as those derived from olive oil, may help reduce oxidative stress in oral tissues and maintain mucosal integrity. In this regard, a recent study showed that green tea extract (GTE) exhibited both anti-inflammatory and wound healing potential in human gingival epithelial keratinocytes (HGEK), indicating its potential as a novel oral anti-inflammatory agent [20,21]. Pinoresinol, one of the simplest lignans, is a dimer of coniferyl alcohol and is commonly found in woody or fibrous plants [22]. Pinoresinol is known to lower the risk of diseases such as breast, prostate, colon, and cardiovascular diseases due to its free radical scavenging activity, antibacterial effects, and anti-inflammatory properties [23]. Its anti-inflammatory properties suggest a potential role in modulating inflammatory processes in periodontal tissues, as inflammation is a key factor in the progression of periodontal diseases. By reducing pro-inflammatory effects in oral tissues and promoting epithelial wound healing, pinoresinol shows therapeutic potential in periodontal health [20,21]. Despite its well-documented systemic benefits, the effects of pinoresinol on oral permeability, epithelial barrier properties, and related gene expression, particularly concerning barrier function and inflammation, have not been extensively studied. In this study, we investigated the effect of pinoresinol on the oral buccal epithelium using a TR146 cell culture model, representing normal human buccal epithelium. Specifically, this study examined the effects of pinoresinol on the expression of TJ-related genes and epithelial integrity by measuring its effects on fluorescein isothiocyanate-dextran 4000 Da (FD4) permeability and transepithelial electrical resistance (TEER).

## Methods

### Materials

Pinoresinol, FD4, dimethyl sulfoxide (DMSO), and Ham’s F-12 medium were purchased from Sigma-Aldrich (St. Louis, MO, USA). Fetal bovine serum (FBS) was obtained from Hyclone Laboratories Inc. (Logan, UT, USA). Penicillin–streptomycin, 0.25% trypsin/EDTA, and 2 mM glutamine were purchased from GIBCO (Gaithersburg, MD, USA). All chemicals and reagents used in this study were of analytical grade and employed without further purification.

### Cell culture

TR146 cell lines (Public Health England, London, UK) were cultured at 37°C in a 5% CO_2_ atmosphere using Ham’s F-12 medium supplemented with 10% FBS and 100 U/mL penicillin-100 µg/mL streptomycin. The cells were maintained in 100 mm culture dishes and dissociated with 0.25% trypsin-EDTA upon reaching 70–80% confluence. The medium was refreshed every two days, and cells between passages 12–20 were used for experiments.

### Cell viability

TR146 cells were seeded into 96-well plates at a density of 5 × 10^4^ cells/well and incubated for 24 h. They were then treated with pinoresinol (0–100 μM) and incubated for another 24 h. Subsequently, the cells were incubated for 1 h at 37°C, followed by the addition of 10 μL of cell counting kit-8 (CCK-8) reagent (Dojindo Laboratories, Kumamoto, Japan) to each well. Cell viability was determined by measuring the optical density (OD) value of each well at a wavelength of 450 nm using a microplate reader (SpectraMax M3; Molecular Devices, San Jose, CA, USA).

### Assessment of FD4 transport and permeability assay

To assess the transport of FD4, TR146 cells (5 × 10^4^ cells/well) were cultured in cell culture inserts (Falcon, Corning Inc., Corning, NY, USA) equipped with a polyethylene terephthalate (PET) membrane (0.9 cm^2^ growth area and 0.4 μm pore size). The TR146 cells seeded in the transwell insert were cultured for 21–28 days, with medium changes every 2–3 days. To investigate the concentration-dependent effects of pinoresinol, TR146 cells were treated with Ham’s F-12 medium containing 10% FBS and various concentrations of pinoresinol (5, 10, 20, and 50 μM) for 24 h at 37°C in a 5% CO_2_ atmosphere. For the time-dependent experiment, cells were treated with 20 μM pinoresinol for 1, 3, 6, and 24 h under the same culture conditions. The medium containing pinoresinol was prepared by diluting a stock solution of each sample, dissolved in DMSO, with a final DMSO concentration of 0.5% in the experiments to avoid cell damage [24]. After incubation, the TR146 monolayer on the transwell insert was gently rinsed with Hanks’ Balanced Salt Solution (HBSS) buffer to remove all compounds. HBSS buffer was then added to both the apical and basolateral sides and stabilized in a 37°C incubator for 10 min. The transport experiments commenced by replacing the apical buffer with fresh HBSS buffer containing FD4 fluorescein (1.0 mg/mL). At 0.5, 1, 1.5, 2, 3, and 4 h, 100 μL samples were drawn from the basolateral compartment (1.5 mL) and replaced with an equal volume of HBSS buffer (pH 7.4) to maintain a constant volume. The fluorescence of the collected samples was measured at an excitation wavelength of 485 nm and an emission wavelength of 535 nm using a fluorescence spectrophotometer (Thermo Fisher Scientific, Waltham, MA, USA). The apparent permeability coefficient (P_app_) was calculated using the following Equation (1):


$$P_{app}(cm/s)=\frac{V}{AC_{0}}\frac{dC}{dt}\;$$ (1)

where V is the assay solution volume in the basolateral compartment (1.5 mL), A is the surface area of the membrane (0.9 cm^2^), C₀ is the initial concentration in the apical compartment (mmol), and dC/dt is the change in concentration in the basolateral compartment over time (mmol/s). The relative P_app_ of fluorescein in TR146 cell monolayers pretreated with the test compound (pinoresinol) was expressed as a percentage (%) of the control P_app._

### TEER measurements

To assess the integrity of TR146 cell layers in permeability experiments, TEER values were continuously monitored. Cells were seeded at a density of 1 × 10^5^ cells/well onto permeable support inserts (Falcon, Corning Inc., USA) equipped with a PET membrane (0.9 cm^2^ growth area and 0.4 μm pore size). TR146 cells between passages 12 and 20 were used for transwell experiments, and the medium was replaced every 2−3 days until monolayer formation (21−28 days). The pinoresinol treatment followed the same protocol as described previously. TEER values were measured immediately after completion of the designated pinoresinol treatment period. TEER values, calculated based on resistance (R in Ω), were measured using an Epithelial Voltohmmeter (EVOM 2, World Precision Instruments, Sarasota, FL, USA) equipped with an STX-2 electrode, according to the manufacturer's instructions. TEER values were calculated using the following Equation (2):


$$\text{TEER }=\;(R_{\left(insert\;with\;cells\right)}\;–\;R_{\left(insert\;without\;cells\right)})\;\times \;A$$ (2)

where *R*_(insert with cells)_ is the resistance (Ω) of the insert with cells; *R*_(insert without cells)_ is the resistance (Ω) of the cell-free insert; and *A* is the surface area of the membrane (0.9 cm²) of the filter.

### Real-time reverse transcription PCR

To assess the effects of pinoresinol on mRNA expression of TJ-related genes, TR146 cells were seeded at a density of 2 × 10^5^ cells/well in six-well plate inserts (PET membrane, 4.2 cm^2^ growth area, 0.4 μm pore size; Falcon, Corning Inc., USA) and cultured for 21−28 days until monolayer formation. Total RNA was extracted from the cells using the RNeasy Mini Kit (Qiagen, Hilden, Germany) following the manufacturer’s protocol. RNA concentration was determined using a NanoDrop™ 2000 spectrophotometer (Thermo Fisher Scientific). For reverse transcription reactions, 1 μg aliquots of total RNA were used as templates, and cDNA synthesis was carried out using the AccuPower PCR Premix (Bioneer, Daejeon, Republic of Korea) and the SimpliAmp Thermal Cycler (Applied Biosystems, Thermo Fisher Scientific). The resulting cDNA was amplified in a 20 μL reaction volume, which included 10 μL of Power SYBR Green PCR Master Mix (Applied Biosystems, Thermo Fisher Scientific), 2 μM of specific primers (Table 1) for TJ-related genes (*occludin*, *claudin-1*, *claudin-3*, *claudin-4, JAM-1*, *MarvelD3*, and *ZO-2*), and *β-actin* (used as an internal control). Sterile deionized water (5.2 μL) completed the reaction mix. PCR amplifications were carried out using a QuantStudio 3 (Applied Biosystems, Thermo Fisher Scientific), starting with a hot start at 95°C for 10 min, followed by 40 cycles of denaturation at 95°C for 15 s, annealing at 60°C for 60 s, and extension at 72°C for 15 s. The expression values of the selected genes were normalized to *β-actin*, with the relative expression level in untreated cells set to 1. All experiments were performed in triplicate.

**Table 1:** Sequences of primers used for real-time reverse transcription PCR (RT-PCR).

Gene name	Primer sequence (5, −3)
*ZO-2*	F: GCTTTGGTGTGGACCAAGAT
	R: TCCATTATGGGTTTGCATGA
*Occludin*	F: TGGCTGCTGCTGATGAATA
	R: CATCCTCTTGATGTGCGATAAT
*MarvelD3*	F: GAACCCCCTTCGGAGAGATA
	R: CGGCAAGGACAAAGTAGGAG
*JAM-1*	F: CTGATCTTTGACCCCGTGAC
	R: ACCAGACGCCAAAAATCAAG
*Claudin-1*	F: ACCGCTCAGGCCATCTAC
	R: CCAGCAGGATGCCAATTAC
*Claudin-3*	F: GAGATGGGAGCTGGGTTGTA
	R: GGATCTTGGTGGGTGCATAC
*Claudin-4*	F: GCTGGGAAGGGCAGTAGAG
	R: ACCAGACGCCAAAAATCAAG
*β-actin*	F: TGTTACCAACTGGGACGACA
	R: AAGGAAGGCTGGAAAAGAGC

### Western blot analysis

For western blot analysis, TR146 cells were cultured under the same conditions as those described above for real-time reverse transcription PCR. The expression levels of TJ-related proteins (occludin, MarvelD3, claudin-1, and claudin-3) in TR146 cells treated with or without pinoresinol for 24 h were estimated using western blotting. Briefly, proteins were extracted using a radioimmunoprecipitation assay (RIPA) buffer (Sigma-Aldrich), supplemented with protease inhibitors (PhosSTOP; Roche, Basel, Switzerland). The total protein concentration per sample was determined using the BCA protein assay kit (Thermo Fisher Scientific). Subsequently, equal amounts of protein (12 μg per lane) were loaded onto 4–12% Bis-Tris Bolt gels (Invitrogen) for electrophoretic separation, followed by transfer onto a polyvinylidene difluoride (PVDF) membrane (Invitrogen). The membrane was then blocked with 5% non-fat dried milk in Tris-buffered saline containing 0.05% Tween 20 (TBS-T) for 1 h at room temperature. Afterward, the membrane was incubated with primary antibodies against occludin (1:1000, Abcam, Waltham, MA, USA), MarvelD3 (1:1000, Proteintech, Rosemont, IL, USA), claudin-1 (1:1000, Invitrogen), claudin-3 (1:1000, Invitrogen), or β-actin (1:2000, Abcam) overnight at 4°C. The membranes were then incubated with secondary antibodies (HRP-conjugated goat anti-rabbit or HRP-conjugated horse anti-mouse IgG; 1:3000, Cell Signaling Technology, Danvers, MA, USA) for 1 h at room temperature. The membranes were subjected to UVP ChemStudio (Analytik Jena, Germany) for western imaging to detect the immunoreactive proteins. The TJ-related proteins were quantified using ImageJ software (NIH, Bethesda, MD, USA). The expression of cellular protein levels was normalized to β-actin, and the normalized results were expressed as protein levels in pinoresinol-treated cells relative to those in control cells without pinoresinol treatment.

### Immunofluorescence

TR146 cells used for immunofluorescence analysis were cultured under the same conditions as previously described for real-time reverse transcription PCR and western blot analysis. Cells were treated with or without 20 μM pinoresinol for 24 h prior to fixation and staining. Cells were fixed with 4% paraformaldehyde (Biosesang, Seongnam, Republic of Korea) for occludin, claudin-1, and claudin-3 for 15 min or cold ethanol (−20°C) for MarvelD3 for 10 min. The samples were then washed three times with PBS and permeabilized with 0.1% Triton X-100 (Duksan, Ansan, Republic of Korea) in PBS for 15 min. After an additional PBS wash, the membranes were blocked with 5% bovine serum albumin (BSA; Sigma-Aldrich, St. Louis, MO, USA) for 1 h. The membranes containing the cell monolayers were then cut using a scalpel and incubated overnight at 4°C with primary antibodies (1:100 for occludin, claudin-1, claudin-3 and MarvelD3) diluted in 1% BSA. The membranes were then washed with PBS and incubated with Goat anti-rabbit IgG (H + L) conjugated with Alexa Fluor 488 (1:1000; Invitrogen) for 1 h. The stained membranes were then mounted on glass slides with an 18 mm × 18 mm coverslip. Fluorescence images were acquired using a confocal laser scanning microscope (LSM 800; Carl Zeiss, Oberkochen, Germany) equipped with a 40 × water-immersion objective and a standard green fluorescence filter, at a resolution of 1024 × 1024 pixels. Z-stack images were captured with a step size of 0.48 µm, and maximum intensity projections were generated for analysis. Image processing and analysis were performed using ZEN Blue software (Carl Zeiss, Germany)

### Statistical analysis

Statistical analyses were conducted using IBM SPSS Statistics for Windows, version 21 (IBM Corp., Armonk, NY, USA). Data are presented as the mean ± standard error of the mean (SEM). To assess statistical significance between groups, a one-way ANOVA was initially performed, followed by the Tukey–Kramer post hoc test with a significance level of *p* < 0.05 to compare differences among mean values. Other statistical evaluations were performed using Student’s *t*-test, also with a significance level of *p* < 0.05.

## Results

### Cytotoxicity of pinoresinol on TR146 cells

**Fig 1:**
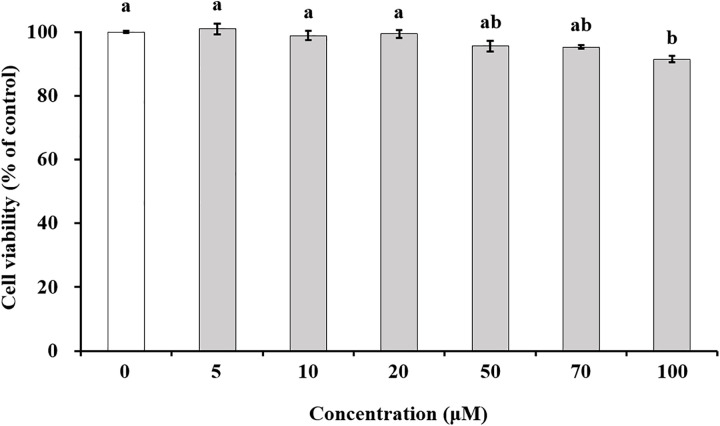
Effect of pinoresinol on TR146 cell viability. TR146 cells were treated with pinoresinol at concentrations of 0 (control), 5, 10, 20, 50, 70, and 100 μM for 24 h, and cell viability was assessed using a CCK-8 assay. Data are presented as the mean ± SEM (n = 6). Lowercase letters indicate statistically significant differences between groups (*p* < 0.05), determined by one-way ANOVA followed by the Tukey-Kramer post hoc test. Groups that do not share the same letter are significantly different.

The cytotoxicity assessment using the CCK-8 assay revealed that TR146 cells treated with 100 μM pinoresinol exhibited significantly lower cell viability compared to the untreated control cells (Fig 1). Consequently, further studies were conducted using non-toxic concentrations of pinoresinol.

### Effect of pinoresinol concentration on TJ barrier integrity and FD4 transport across TR146 cell monolayers

**Fig 2:**
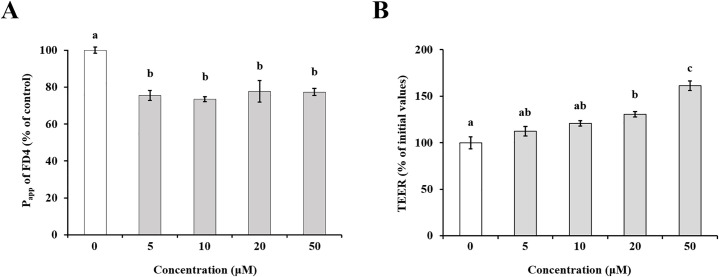
Effect of pinoresinol concentration on TR146 cell monolayers. TR146 cells were pretreated with pinoresinol at concentrations of 0, 5, 10, 20, and 50 μM for 24 h. (A) Apparent permeability (P_app_) values of FD4 and (B) TEER values of TR146 cell monolayers treated with or without pinoresinol. Data are presented as the mean ± SEM (n = 4). Lowercase letters indicate statistically significant differences between groups (*p* < 0.05), determined by one-way ANOVA followed by the Tukey-Kramer post hoc test. Groups that do not share the same letter are significantly different.

To confirm the concentration-dependent effects of pinoresinol on TR146 cell monolayers, fluorescein flux and TEER values were measured. TR146 cell monolayers were treated with various concentrations of pinoresinol (5, 10, 20, and 50 μM) for 24 h. As shown in Fig 2A, the P_app_ of FD4 was significantly decreased in cell monolayers treated with pinoresinol at concentrations of 5 μM or higher (Control: 1.27 ± 0.02 × 10^-3^ cm·s^-1^; 5 μM: 0.96 ± 0.03 × 10^-3^ cm·s^-1^; 10 μM: 0.93 ± 0.02 × 10^-3^ m·s^-1^; 20 μM: 0.99 ± 0.07 × 10^-3^ cm·s^-1^; 50 μM: 0.98 ± 0.02 × 10^-3^ cm·s^-1^). Additionally, TEER values were significantly increased in cell monolayers treated with pinoresinol at 20 μM or higher (Control: 78 ± 5.08 Ω·cm^2^; 5 μM: 88 ± 3.98 Ω·cm^2^; 10 μM: 94 ± 2.31 Ω·cm^2^; 20 μM: 102 ± 2.33 Ω·cm^2^; 50 μM: 126 ± 4.04 Ω·cm^2^, Fig 2B) compared to untreated cells. Based on these results, 20 μM pinoresinol was selected to evaluate its effects on barrier integrity and FD4 transport to elucidate the time-dependent manner.

### Effect of pinoresinol treatment time on TJ barrier integrity and FD4 transport across TR146 cell monolayers

**Fig 3:**
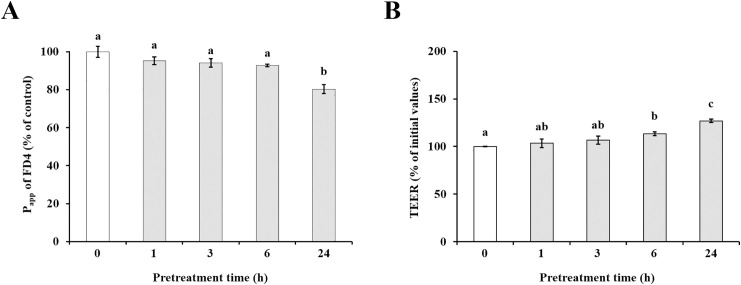
Effect of pinoresinol treatment time on TR146 cell monolayers. TR146 cells were pretreated with 20 μM pinoresinol for 1, 3, 6, and 24 h. (A) Apparent permeability (P_app_) values of FD4 and (B) TEER values of TR146 cell monolayers treated with or without pinoresinol. Data are presented as the mean ± SEM (n = 4). Lowercase letters indicate statistically significant differences between groups (*p* < 0.05), determined by one-way ANOVA followed by the Tukey-Kramer post hoc test. Groups that do not share the same letter are significantly different.

Based on the concentration results, 20 μM was selected as the minimum effective dose for subsequent time-course experiments. To investigate the time-dependent effects of pinoresinol, fluorescein flux and TEER were measured in TR146 cell monolayers pretreated with 20 μM pinoresinol for 1, 3, 6, and 24 h. As shown in Fig 3A, the apparent permeability of fluorescein was significantly reduced after 24 h of treatment (Control: 1.60 ± 0.05 × 10^-3^ cm·s^-1^; 1 h: 1.52 ± 0.03 × 10^-3^ cm·s^-1^; 3 h: 1.51 ± 0.03 × 10^-3^ cm·s^-1^; 6 h: 1.49 ± 0.01 × 10^-3^ cm·s^-1^; 24 h: 1.29 ± 0.04 × 10^-3^ cm·s^-1^). TEER values were significantly increased in cells treated for 6 h or longer, compared to untreated cells (Control: 73 ± 0.32 Ω·cm^2^; 1 h: 76 ± 3.17 Ω·cm^2^; 3 h: 78 ± 3.07 Ω·cm^2^; 6 h: 83 ± 1.51 Ω·cm^2^; 24 h: 93 ± 1.40 Ω·cm^2^, Fig 3B).

### Effect of pinoresinol on the expression of TJ-related genes in TR146 cell monolayers

**Fig 4:**
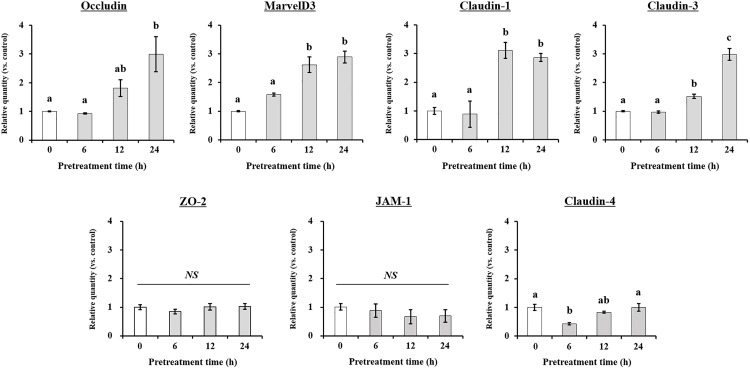
Effect of pinoresinol on mRNA expression of TJ-related genes in TR146 cells. TR146 cells were treated with 20 μM pinoresinol for 6, 12, and 24 h, and the expression of mRNAs was compared with untreated cells. Total RNA was extracted, and mRNA expression levels were assessed using real-time reverse transcription PCR with specific primers targeting *occludin*, *JAM-1*, *MarvelD3*, *claudin-1*, *claudin-3*, *claudin-4*, *ZO-2*, and *β-actin*. Data are presented as the mean ± SEM (n = 3). Lowercase letters indicate statistically significant differences between groups (*p* < 0.05), and “NS” indicates no significant difference between groups, determined by one-way ANOVA followed by the Tukey-Kramer post hoc test. Groups that do not share the same letter are significantly different.

**Fig 5:**
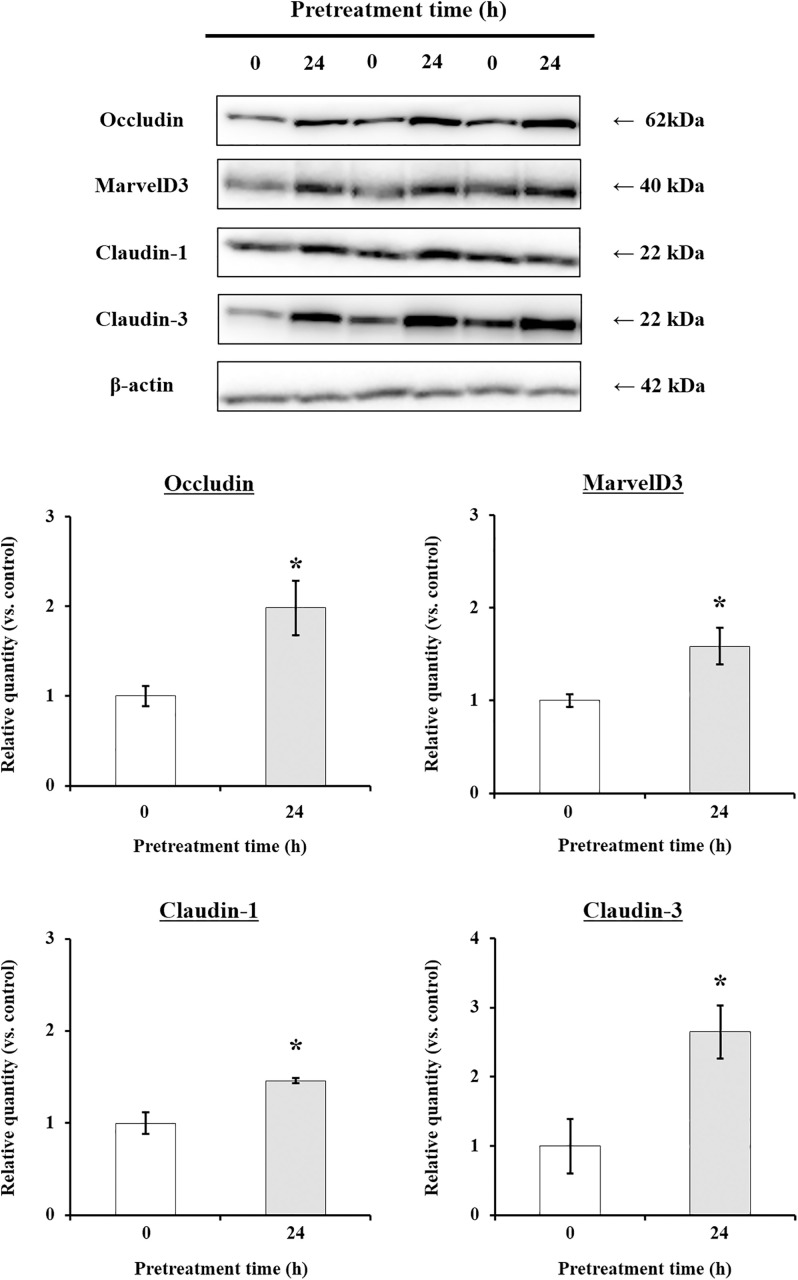
Effect of pinoresinol on the expression of TJ-related proteins in TR146 cells. TR146 cells were treated with 20 μM pinoresinol for 24 h, and the expression of proteins was compared with untreated cells. Specific bands corresponding to occludin, MarvelD3, claudin-1, claudin-3, and β-actin were quantified using densitometric analysis. Data are presented as the mean ± SEM (n = 3). (*) *p* < 0.05 indicate significant differences from untreated cells. Significant differences between the untreated (control) and 20 μM pinoresinol-treated groups were evaluated using the unpaired Student’s *t*-*t*est.

**Fig 6:**
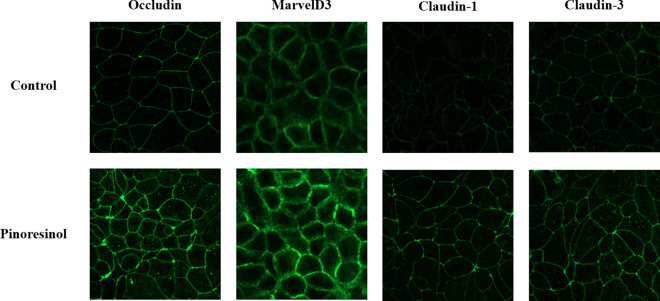
Effect of pinoresinol on TJ structure in TR146 cells observed by confocal microscopy. TR146 cells were treated with 20 μM pinoresinol for 24 h, and the fluorescence intensity of TJ-related targets, including occludin, MarvelD3, claudin-1, and claudin-3, were analyzed using immunofluorescence microscopy. The fluorescence signals were quantified and compared with those of untreated cells.

Next, to confirm the mechanisms by which pinoresinol affects barrier function, TR146 cells were pretreated with 20 μM pinoresinol for 6 h or longer. Assessment of the mRNA and protein expression levels of TJ-related genes revealed that the mRNA expression levels of MarvelD3, claudin-1, and claudin-3 were significantly increased after 12 h of pinoresinol treatment (Fig 4). Moreover, the mRNA expression levels of occludin, MarvelD3, claudin-1, and claudin-3 were significantly higher in pinoresinol-treated cells compared to control cells, whereas the expression levels of JAM-1, claudin-4, and ZO-2 showed no significant increase. Consistently, pinoresinol-treated TR146 cells exhibited significantly higher protein levels of occludin, MarvelD3, claudin-1, and claudin-3 than untreated control cells (Fig 5), suggesting that pinoresinol effectively enhanced the expression of TJ-related genes. Supporting these findings, immunofluorescence analysis also confirmed a marked increase in the junctional localization of occludin, MarvelD3, claudin-1, and claudin-3 in the pinoresinol-treated group compared to the control group (Fig 6).

## Discussion

The oral mucosa epithelium, composed primarily of non-keratinized squamous cells, is continuously exposed to microorganisms, antigens, and environmental stimuli [25]. Its barrier integrity, maintained by TJs, is essential for protecting underlying tissues from pathogen infiltration and inflammatory insults [26]. Disruption of TJs has been implicated in several oral pathologies such as oral mucositis, periodontitis, and oral squamous cell carcinoma (OSCC) [27–29], highlighting the importance of restoring epithelial barrier function in disease prevention and therapy. TJ proteins, including occludin, MarvelD3, and claudins, are critical for maintaining the selective permeability of the epithelial barrier [30,31]. Disruption or altered expression of these proteins increases paracellular permeability and initiates inflammatory cascades. Several studies have demonstrated that modulation of TJ-related protein expression can influence barrier function, and natural compounds, particularly polyphenols, have emerged as potential TJ modulators [32–35]. Suzuki *et al*. demonstrated that kaempferol promotes barrier function through occludin, claudin-1, and claudin-3 accumulate at cell-cell junctions [33]. Park and Yu also showed the modulation of TJ-related proteins including occludin, MarvelD3, JAM-1, and claudin-1 by hesperidin [34]. It has been reported that theaflavin enhanced the intestinal barrier through the expression of occludin, claudin-1, and ZO-1 [35]. Although, effect of some of polyphenols of the natural bioactive lignan family such as phillygenin on TJ-related gene modulation in mice chronic colitis were demonstrated [36], investigations of pinoresinol on epithelial barrier function, especially oral, remain limited. In this study, we demonstrated that pinoresinol enhances the barrier integrity and function of human buccal epithelial cells using a well-established TR146 monolayer model. TR146 cells cultured for 3–4 weeks on permeable supports recapitulate key features of the non-keratinized oral epithelium and suitable for evaluating epithelial permeability and barrier dynamics [18,37–40]. Pinoresinol treatment at non-toxic concentrations (Figs 1 and S1) significantly reduced FD4 permeability and increased TEER values (Figs 2, 3, S2 and S3), indicating strengthened barrier integrity and reduced paracellular transport. These results suggest that pinoresinol primarily modulates the TJ-mediated route of permeability, as TEER is a widely accepted indicator of TJ integrity [41–43]. TEER values reflect the degree of ionic conductance across cell monolayers and are closely associated with the function of TJ-related proteins [42,43]. Numerous studies have emphasized that TJ-associated proteins such as occludin, MarvelD3, and claudins play a crucial role in both structural assembly and permeability regulation [34–37,44,45]. Consistent with these findings, we observed that pinoresinol selectively upregulated the gene expression of occludin, MarvelD3, claudin-1, and claudin-3 in Figs 4 and 5. However, the expression mRNA levels of JAM-1, claudin-4, and ZO-2 showed no significant increase. Although claudin-4 showed a transient decrease of mRNA at 6 h, its expression returned to control levels after 12 h (Fig 4). This specificity suggests that pinoresinol acts on distinct subsets of TJ components, rather than exerting a general upregulation across all TJ-related genes. Furthermore, immunofluorescence analysis confirmed enhanced localization of these proteins at intercellular junctions (Figs 6 and S4), which is a critical determinant of functional TJ assembly. Similar to our observations, Lin *et al.* demonstrated that overexpression of occludin and claudin-1 increased TEER and reduced permeability in vitro barrier model of the human submandibular salivary gland epithelium [46]. Naylor *et al.* reported that marvelD3 plays a structural role in maintaining TJ architecture, highlighting its potential as a barrier-regulating target [47]. It was also shown that MarvelD3, claudin-1, and claudin-3 act as key factor for enhanced oral barrier integrity and function by Park *et al*. [48]. In light of these findings, pinoresinol appears to enhance TJ integrity through protein-specific regulatory mechanisms. Notably, previous studies have indicated that TJ modulation is often protein-specific. Previous studies have also shown that post-translational modifications of TJ-related proteins, such as phosphorylation of claudin-1 and occludin, are differentially regulated by mitogen-activated protein kinase (MAPK) and protein kinase C (PKC) pathways, affecting their junctional function [49,50]. These observations underscore the importance of characterizing the molecular selectivity of candidate compounds like pinoresinol. Importantly, the upregulation and membrane localization of occludin, MarvelD3, claudin-1, and claudin-3 observed in this study supports their synergistic role in reinforcing epithelial barrier [33–35,44,45]. Our findings thus expand the repertoire of polyphenols with oral barrier protective effects and suggest that pinoresinol may be a promising agent for preventing or alleviating TJ-disruptive oral conditions. Such conditions, including periodontitis and mucositis, are often associated with increased permeability and inflammatory responses. By stabilizing TJ structures, pinoresinol could provide protective and restorative benefits. Moreover, the use of pinoresinol may complement existing therapies by reducing tissue susceptibility to inflammation-driven damage. To fully elucidate the therapeutic potential of pinoresinol, future studies should explore its effects in inflammation-induced models of oral epithelial dysfunction. In particular, its influence on pro-inflammatory cytokine expression and tissue repair dynamics should be investigated. Mechanistic studies focusing on signaling pathways for TJ protein stability and cytoskeletal interactions such as Toll-like receptor 4 (TLR4)/ nuclear factor kappa B (NF-κB), AMP-activated protein kinase (AMPK), MAPK, and phosphoinositide3-kinase (PI3K)/Akt may reveal the underlying molecular basis of pinoresinol’s action [32,51–53]. In conclusion, this study provides novel evidence that pinoresinol reinforces oral epithelial barrier integrity by modulating the expression and localization of key TJ-related proteins. These findings support the potential of pinoresinol as a natural agent in the prevention and management of oral diseases associated with TJ disruption. By demonstrating protein-specific enhancement of TJ integrity, this study provides a mechanistic foundation for future translational research aiming to utilize pinoresinol in the prevention and treatment of barrier-compromised oral conditions.

## Conclusions

This study demonstrates that pinoresinol significantly enhances the integrity and function of the oral epithelial barrier. It increases TEER, reduces the paracellular permeability, and upregulates the expression of TJ-associated molecules such as occludin, MarvelD3, claudin-1, and claudin-3. Pinoresinol also promotes their junctional localization, further supporting its role in strengthening barrier function. These findings suggest that pinoresinol may have potential therapeutic applications in preventing and managing periodontal diseases by protecting against epithelial barrier dysfunction caused by inflammation or mechanical damage. By reinforcing epithelial defenses, pinoresinol offers promise for integration into oral care strategies, such as mouthwashes or topical formulations, to support periodontal health. Its ability to regulate TJ pathways highlights its promise as a novel agent in maintaining periodontal health.

## Supporting information

S1 Fig Effect of pinoresinol on TR146 cell viability. TR146 cells were treated with pinoresinol at concentrations of 0, 5, 10, 20, 50, and 70 μM for 24 h, or 0.1% Triton X-100 detergent (positive control). Cell viability was assessed using a CCK-8 assay. Data are presented as the mean ± SEM (n = 6). “NS” indicates no significant differences between pinoresinol groups (*p* ≥ 0.05), as determined by one-way ANOVA followed by the Tukey-Kramer post hoc test.
(TIF)

S2 Fig Dot plots for Fig 2. (TIF)

S3 Fig Dot plots for Fig 3. (TIF)

S4 Fig Quantitative analysis of the fluorescence intensity by pinoresinol on TJ structure in TR146 cells observed by confocal microscopy. TR146 cells were treated with 20 μM pinoresinol for 24 h, and the fluorescence intensity of TJ-related proteins, including occludin, MarvelD3, claudin-1, and claudin-3, were analyzed by immunofluorescence microscopy. Fluorescence signals were quantified as fluorescence intensity per unit area and compared with those of untreated cells. Data are presented as the mean ± SEM (n = 5). (**) *p* < 0.01 indicate significant differences from untreated cells, as determined by unpaired Student’s *t*-test.
(TIF)

S5 Fig Full blots for Fig 5. (PDF)

S6 Fig Raw data underlying the quantification and analyses presented in Figs 1–5, S1 and S2.
(ZIP)
