# Supplementary figures and images for: Pinoresinol enhances oral barrier integrity and function in human buccal cell monolayers
Source: PLoS One. 2025 Sep 8;20(9):e0331242. doi: 10.1371/journal.pone.0331242 (PMC12416738; doi:10.1371/journal.pone.0331242)

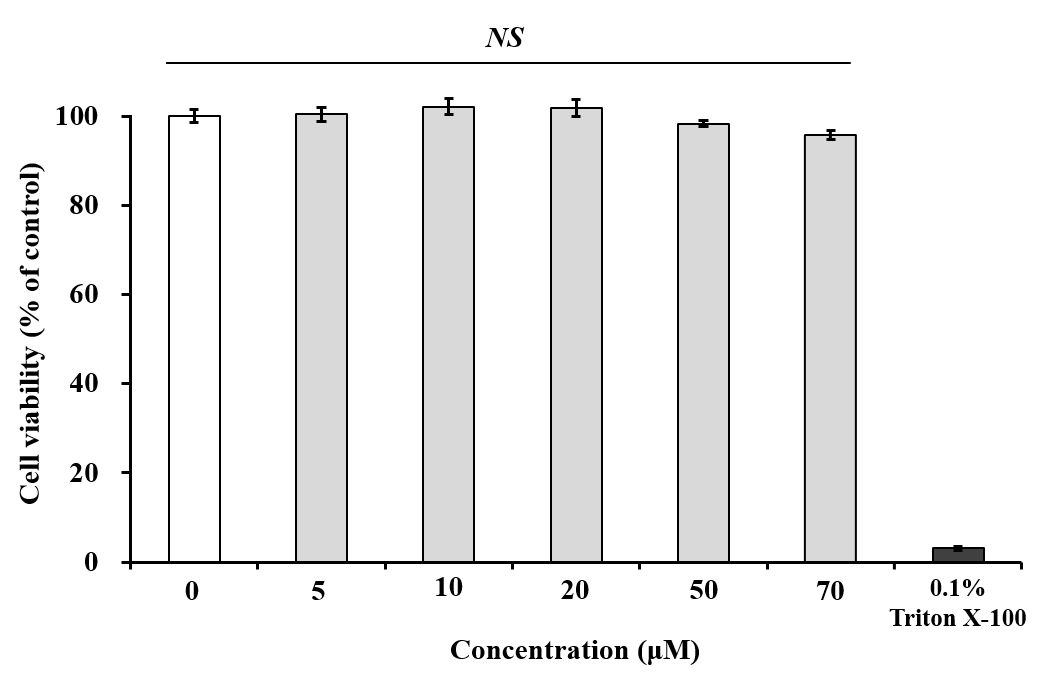

Supplement: S1 Fig — TR146 cells were treated with pinoresinol at concentrations of 0, 5, 10, 20, 50, and 70 μM for 24 h, or 0.1% Triton X-100 detergent (positive control). Cell viability was assessed using a CCK-8 assay. Data are presented as the mean ± SEM (n = 6). “NS” indicates no significant differences between pinoresinol groups (p ≥ 0.05), as determined by one-way ANOVA followed by the Tukey-Kramer post hoc test. (TIF) [file pone.0331242.s001.tif]

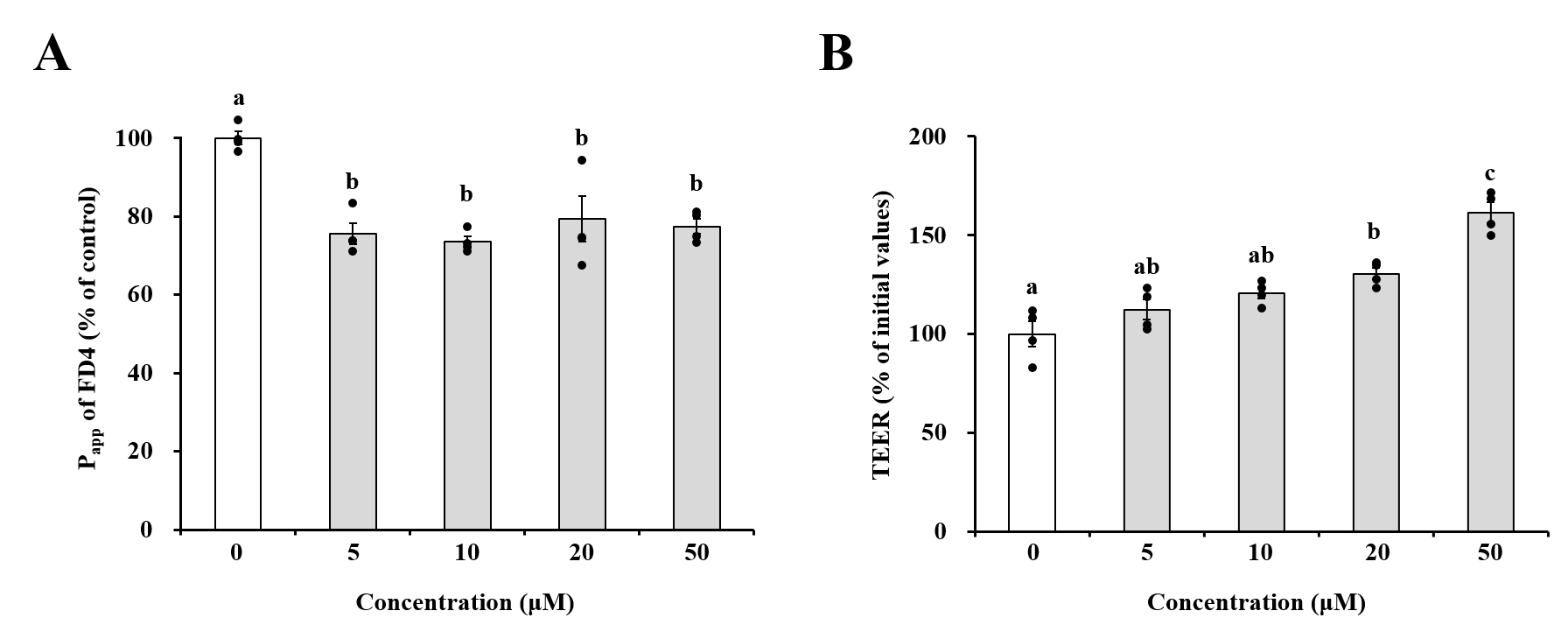

Supplement: S2 Fig — (TIF) [file pone.0331242.s002.tif]

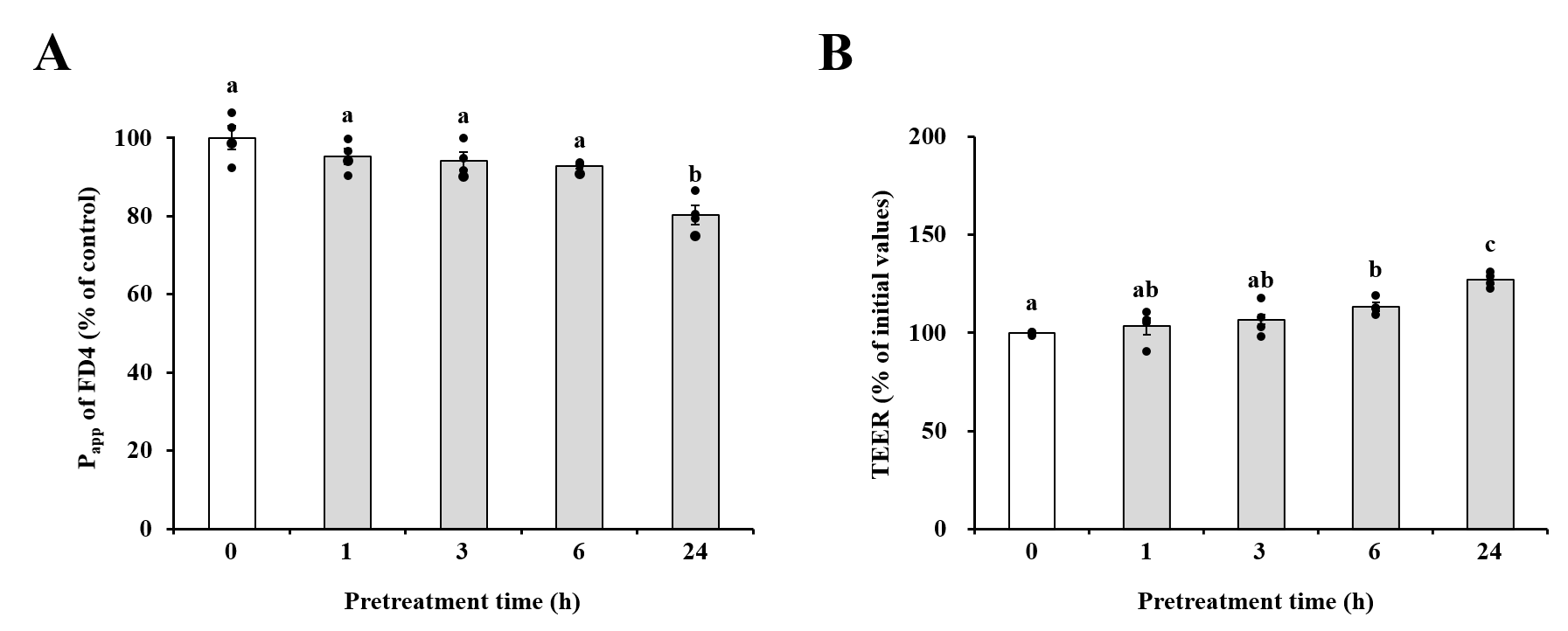

Supplement: S3 Fig — (TIF) [file pone.0331242.s003.tif]

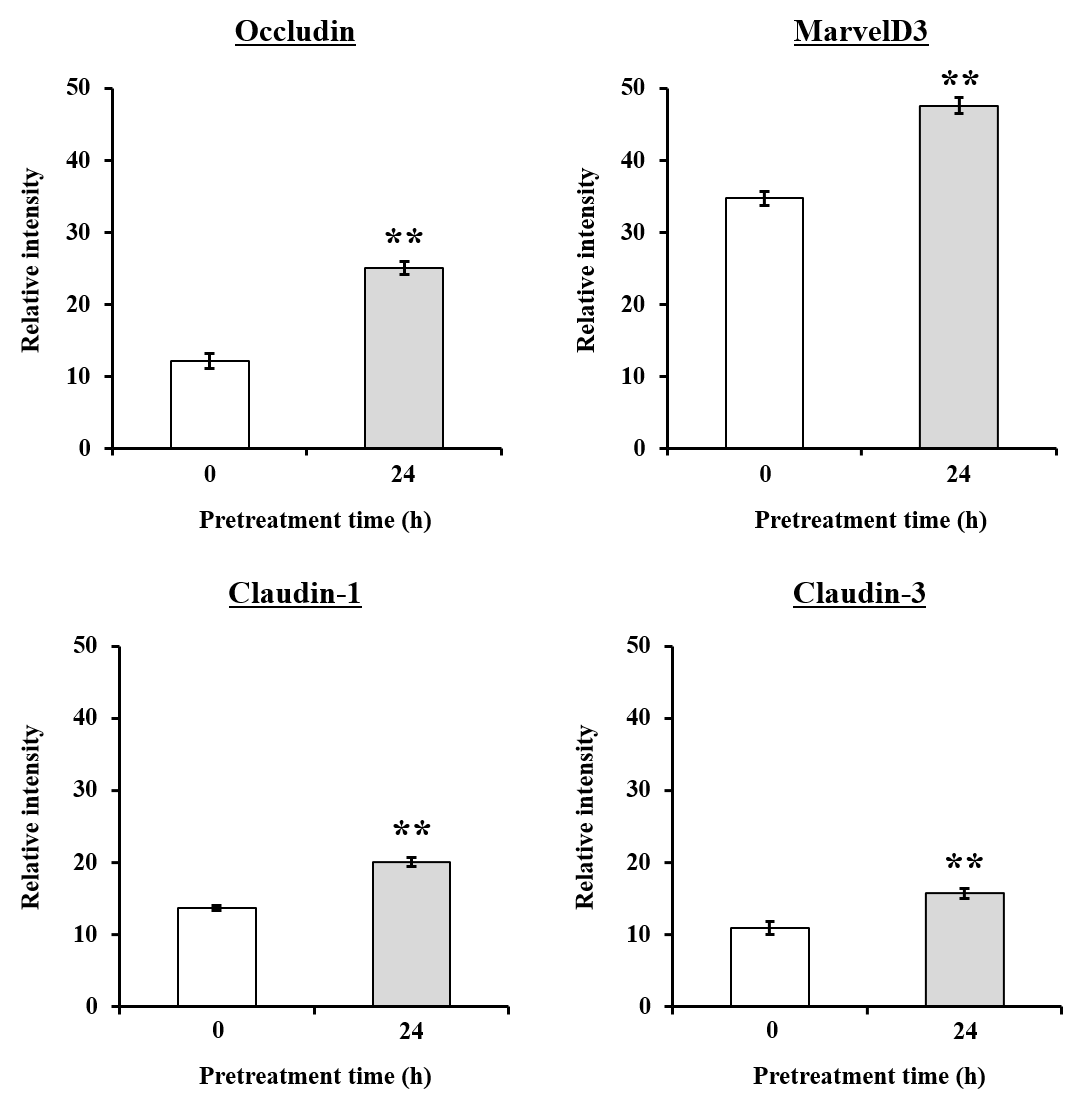

Supplement: S4 Fig — TR146 cells were treated with 20 μM pinoresinol for 24 h, and the fluorescence intensity of TJ-related proteins, including occludin, MarvelD3, claudin-1, and claudin-3, were analyzed by immunofluorescence microscopy. Fluorescence signals were quantified as fluorescence intensity per unit area and compared with those of untreated cells. Data are presented as the mean ± SEM (n = 5). (**) p < 0.01 indicate significant differences from untreated cells, as determined by unpaired Student’s t-test. (TIF) [file pone.0331242.s004.tif]

S5.

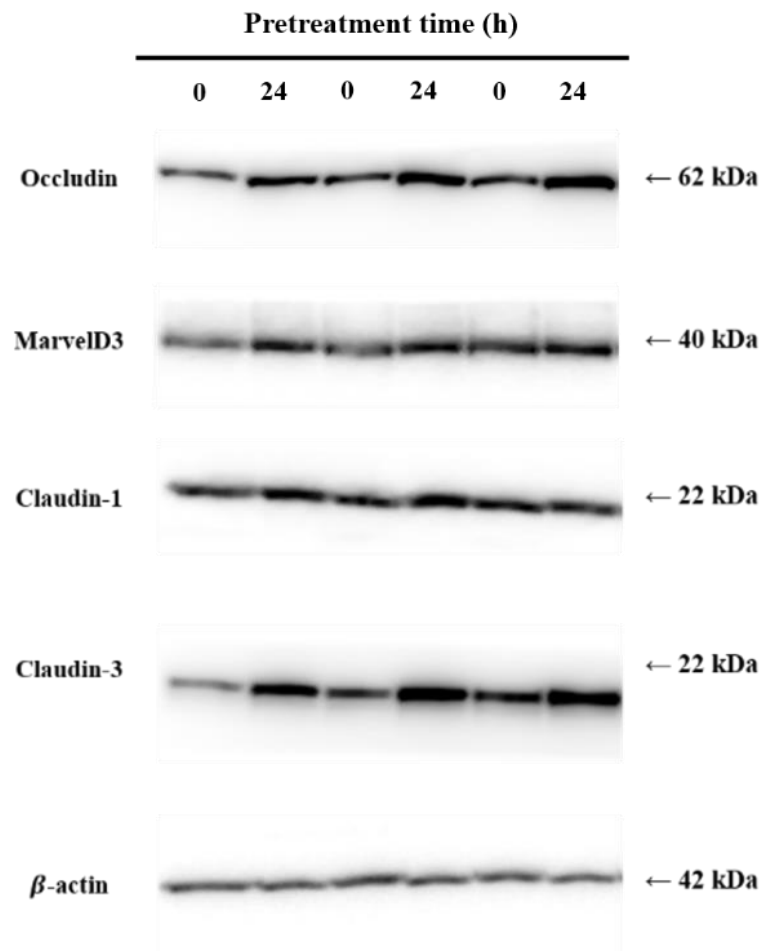

S5. Full blots for Figure 5

Supplement: S5 Fig — (PDF) [file pone.0331242.s005.pdf]
